# Improving Documentation of Pain Reassessment after Pain Management Interventions in the NICU

**DOI:** 10.1097/pq9.0000000000000688

**Published:** 2023-09-28

**Authors:** Smitha Israel, Sofia Perazzo, Morgan Lee, Rachel Samson, Parissa Safari-Ferra, Ranjodh Badh, Solomon Abera, Lamia Soghier

**Affiliations:** From the * Division of Neonatology at Children’s National Hospital; † The Department of Pediatrics at the George Washington University School of Medicine and Health Sciences; ‡ The Neonatal Intensive Care Unit at Children’s National Hospital; § The Quality Improvement and Safety Department at Children’s National Hospital; ¶ Center of Pediatric Informatics at Children’s National Hospital.

## Abstract

**Background:**

Neonates exposed to painful procedures require pain assessment and reassessment using nonverbal scales. Nurses perform initial assessments routinely, but reassessment is variable. The goal was to increase pain reassessments in neonates with a previous score of 4 or higher within 60 minutes from 50% to 75% within 12 months.

**Methods:**

After identifying key drivers, we tested several interventions using the IHI’s Model for Improvement. The outcome measure was the rate of reassessments within 1 hour after scoring ≥4 on the Neonatal Pain Agitation and Sedation Scale (N-PASS). Duration of time between scoring and intervention was documented. Interventions included electronic health record (EHR) changes, direct communication with bedside nurses through text messages and emails, in-person education, and a yearly competency module. The process measure was the number of messages/emails to staff. Sedation scores were the balancing measure.

**Results:**

Baseline compliance was 50% with significant variability. A centerline shift occurred after the first intervention. After the first four interventions in the following 3 months, a 29% total increase occurred. Overall time-lapse between reassessments decreased from 102 to 90 minutes. Overall sedation scores decreased from -2.5 during the baseline to -1.7 during the sustain period. The goal of 75% pain reassessments was achieved and sustained for two years.

**Conclusions:**

Automated tools such as the trigger report provided data that increased noncompliance visibility. Real-time and personalized reminders and education improved awareness and set the tone for culture change. Electronic health record reminders for reassessments and standardized annual education helped in sustaining change.

## INTRODUCTION

Neonates may experience more than three hundred painful procedures and surgeries throughout their hospitalization.^1,2^ Repeated exposure to pain in neonates can result in short- and long-term neurodevelopmental effects, including visual perceptual difficulties at school and maladaptive pain response later in life.^3–7^

Proactive pain assessment allows for early detection and adequate management to minimize its impact. Assessment of pain in nonverbal infants is complex^5^; therefore, clinicians should use standardized tools and reassess periodically, particularly after providing interventions.^8,9^ Reassessment is an essential step in improving pain management as required by The Joint Commission in its standards for hospitals.^10^ The American Academy of Pediatrics also stressed the importance of thorough pain assessment and management in its 2016 policy statement.^4^ In 2018, The Joint Commission issued a revised report on the pain assessment and management standards for accredited hospitals, including neonatal intensive care units. The report noted that hospitals should “*reassess and respond to pain through evaluation and documentation of response to pain intervention(s) promptly”* and that the performance improvement team should collect and analyze this data.^10^ Reassessment and improvement of “pain documentation behavior, especially in the pediatric population,” as described by Margonary et al, remain a gap in improvement science research and clinical practice.^11^

From 2019 to 2021, the NICU participated in a multicenter quality improvement (QI) collaborative through the Children’s Hospitals Neonatal Consortium (CHNC) focused on postoperative pain management. Our unit documented pain reassessments after a high score only 50% of the time because patient care was demanding, and real-time electronic health record (EHR) documentation was challenging. Our staff of approximately 200 nurses continually changes as we onboard new nurses. Although nurses learned pain assessments and documentation during orientation, specific areas, such as pain reassessment, needed reinforcement. The main goal of this QI study was to increase pain reassessment and accurate documentation in the NICU within 60 minutes by 25% within 12 months (October 2020 to October 2021).

## METHODS

### Setting:

Children’s National Hospital is a free-standing children’s hospital that serves the District of Columbia, Virginia, and Maryland. Its neonatal intensive care unit is a 66-bed level-IV nondelivery NICU. The NICU received over 1100 patients during 2021 with complex diagnoses ranging from congenital anomalies requiring surgeries to other genetic, metabolic, and neurologic conditions. One-third of admissions are surgical. The NICU rooms are of a hybrid design composed mostly of single-family rooms with two shared pods of four beds each. The nurse-to-patient ratio is between 2-3 and 1, and the nurses work 12-hour shifts. The unit has always had a strong culture for pain assessment using validated pain scales, including the premature infant pain profile^12^ and, most recently, the Neonatal Pain, Agitation, and Sedation Scale (N-PASS).^13^

### Scale:

The N-PASS comprises two scales, a pain/agitation scale and a sedation scale. The pain/agitation scale ranges from 0 to +10. The sedation scale is a negative scale ranging from 0 to -10.^13^ Light sedation is from -2 to -5, and deep sedation is -5 to -10. Best practice dictates that pain reassessment occurs within one hour when a patient scores ≥4 on the N-PASS.^14,15^ We chose the 60-minute reassessment timeframe to cover most scenarios faced in the NICU, where the most common pharmacological agent used is morphine. Nurses document the N-PASS scores in the EHR, which is accessible to the physician team. The nurse must document and select each scale component from a drop-down menu. The system calculates the final score once the nurse enters all the components. Multidisciplinary morning rounds emphasize reporting the N-PASS daily to alert the team for further pain management. Compared with using the N-PASS in initial scoring (100%), nonpharmacological pain management interventions (100%), and initial documentation, our documentation of pain reassessment was considerably below our desired rates. These data were obtained before the start of this study through our participation in the CHNC ERASE pain collaborative (https://www.thechnc.org/quality-improvement/erase-pain).

### QI team and Improvement Model:

To increase reassessments and improve the accuracy of EHR documentation, we used the IHI model for improvement. A multidisciplinary team consisting of a bedside nurse, a nurse educator, physicians, a pharmacist, and QI specialists participated. This project was undertaken as a QI Initiative and did not constitute human subject research. As such, it did not require oversight by the institutional review board. The SMART aim was to increase the percentage of pain reassessments for neonates with a previous score of ≥4 within 60 minutes from 50% to 75% within 1 year (October 2020 to October 2021).

### Planning the intervention:

**Fig. 1.**
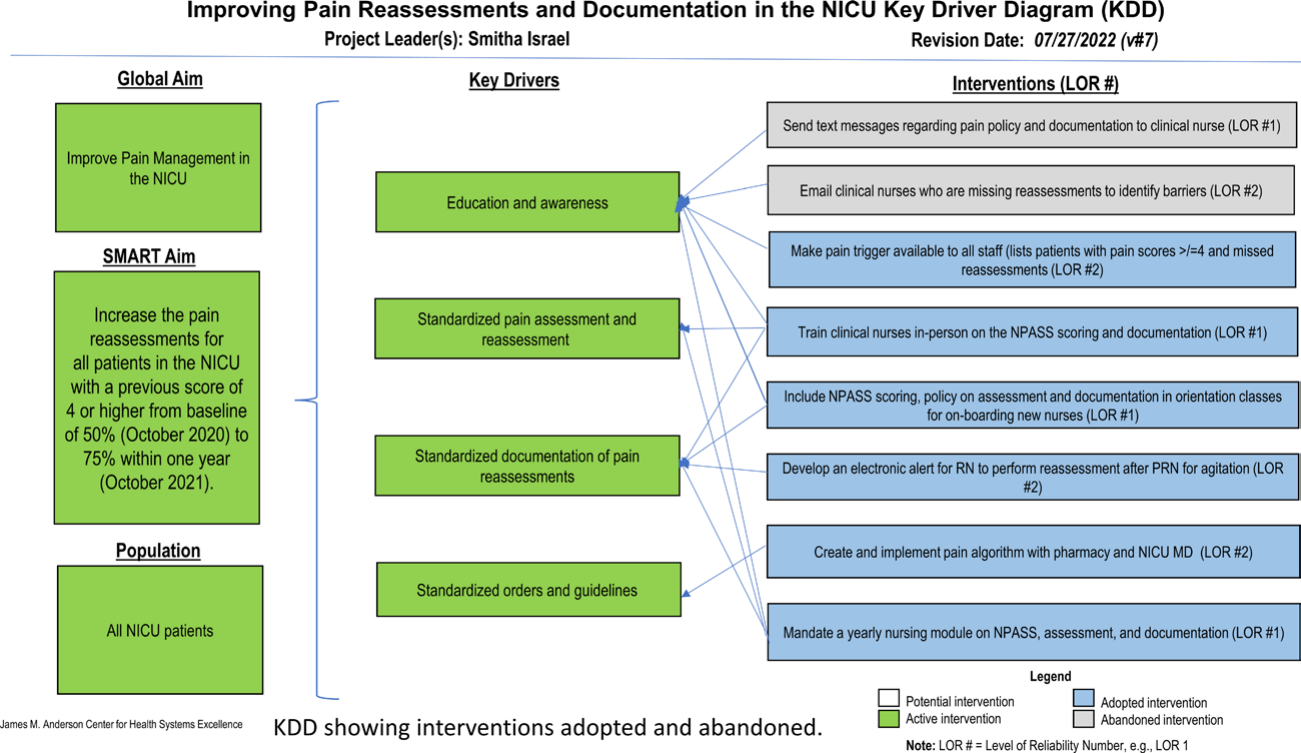
Key driver diagram showing interventions adopted or abandoned.

We composed a key driver diagram (Fig. 1) and performed a failure modes effects and analysis showing the pathway to pain documentation (Supplemental Figure 1, http://links.lww.com/PQ9/A515). We tested interventions using Plan Do Study Act (PDSA) cycles (Table 1).

**Table 1.** Plan-Do-Study-Act Cycles Showing Date and Description of PDSAs

Date of PDSA	Description of PDSA
PDSA #1: 10/14/2020	Text message all clinical nurses regarding pain policy and documentation
PDSA #2: 11/24/2020	Text-message-specific clinical nurses missing pain reassessments
PDSA #3: 12/07/2020	Email clinical nurses who are missing reassessments to identify barriers
PDSA #4: 12/29/2020	Educate clinical nurses in-person
PDSA #5: 8/06/2021	EHR modification to alert RN to perform reassessment when selecting N-PASS agitation after administering PRN for agitation
PDSA #6: 12/1/2021	Mandate yearly module on N-PASS, assessment, reassessment, and documentation

### Measures:

The baseline was collected daily from October 6, 2020 to October 13, 2020, giving eight baseline data points. We determined the numerator by counting the patients with a score of ≥4 and a time-lapse of less than one hour for the next documented pain score in the previous month. The denominator was all patients with a pain score ≥ 4 within the same period. The outcome measure was the percentage of patients with a score of ≥ 4 that had a second reassessment within one hour. The secondary outcome measure was the time between pain assessments when a score of ≥4 was assessed (time-lapse). The balancing measure was the change in sedation scores over the same period. The process measures were the number of texts and emails sent and the number of nurses who received in-person education.

### Interventions:

We based our interventions on increasing staff awareness of the importance of pain assessment and reassessment in the NICU, developing automated reporting tools, including teaching material and policy in new nurses’ orientation, yearly competency assessment, standardization of pain management, and physician discussion on rounds. This period ran from October 14, 2020 to December 31, 2021. First, we texted all nurses to remind them of the policy (PDSA #1). Later we changed this communication reminder to target some selected nurses who cared for a patient with a missing reassessment on manual audit (PDSA #2).

We partnered with the hospital triggers program to create an automated daily report for NICU patients with N-PASS scores ≥4. The hospital triggers program is a unique patient safety program that provides near real-time reporting of harm events, eg, hyaluronidase use for IV infiltrates.^16^ We reprogramed this report to give real-time reporting of pain scores ≥4 and cross-reference missed reassessments. The triggers program extracts data directly from the EHR, but the report is built in HealtheIntent (a data processing platform, Cerner, Kansas City, Mo.). The final report listed the patient’s name, score, time of the score, the intervention of said score, and the time-lapse between scoring and reassessment. During the final cycle of the PDSA ramp, we sent emails to all nurses who missed documentation, as identified by the automated trigger program (PDSA #3), asking about the specific circumstances of the event with the intent of learning about barriers to reassessment and documentation. The nurses noted that standardization of documentation and education were their biggest barriers. Lack of time was another major cause of nurses not completing reassessment documentation.

The self-service report was unavailable to managers. It ran daily with extensive review weekly by the QI team. One ethical consideration was identifying patients who could be suffering from continuous pain. Therefore, it was necessary to alert the care team if this happened. Concomitantly, the nurse educators conducted in-person training (PDSA #4). Each training session lasted approximately 5–8 minutes and included a review of the N-PASS scale, appropriate assessments, reassessments, and documentation.

Subsequently, the team noticed an automated reminder in the EHR to reassess the patient within one hour for the pain portion of the N-PASS but not for the agitation portion. Therefore, we developed an additional automated reminder that prompted the nurse to enter a reassessment for a score ≥4 on the agitation portion (PDSA #5). We, therefore, created a new pathway for documenting medication given due to agitation.

We added a mandatory annual nursing education module (PDSA #6) for nursing staff. It was a refresher course on the N-PASS, how best to use it, how often to assess pain and agitation, and how to document assessments and reassessments. The nurse educators also gave the new nurses education during orientation.

After this final PDSA, we monitored for sustainability from January 1, 2022, through July 30, 2022. We prioritized PDSAs based on priority and ease of implementation (Prioritization Matrix – Supplemental Figure 2, http://links.lww.com/PQ9/A516).

### Analysis:

We developed statistical process control charts to monitor and analyze the data. We considered a centerline shift when it conformed with standard IHI statistical process control chart rules^18^ and analyzed the data using the QI charts Excel add-in (Process Improvement Products, San Antonio, Tex.). We gathered data for compliance on a P-chart from October 2020 to August 2022 and data for the time-lapse on an X-bar S chart from December 2020 to August 2022.

## Results:

**Fig. 2.**
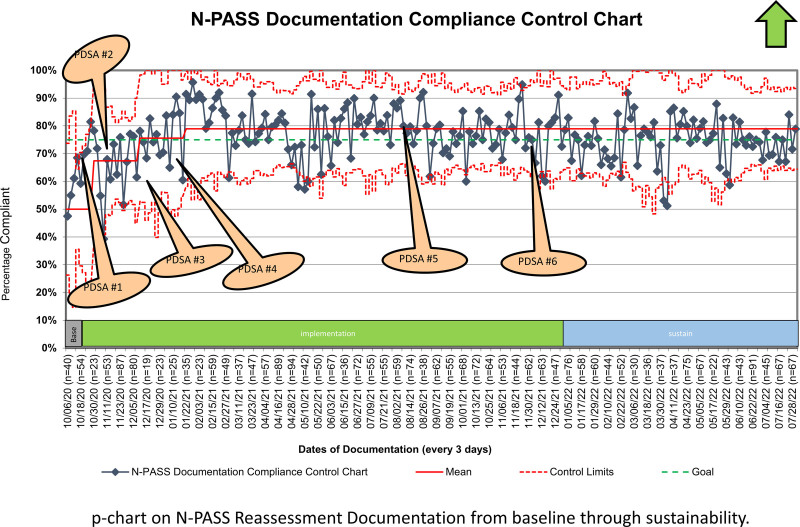
N-PASS documentation compliance chart (p-chart). P-chart on N-PASS reassessment documentation from baseline through sustainability.

Using the trigger tool, we documented 11,263 pain reassessments over 21 months with 1227 unique patients from December 2020 to August 2022. At baseline, nurses reassessed 50% of encounters with patients experiencing high pain scores and large variability. We saw improvements after the initiation of the first four PDSA cycles. The P-chart showed normal variability within control limits during the baseline period and a centerline shift from 50% to a final of 79% (29% increase), exceeding the goal of 75%, and was sustained for the next 6 months (Fig. 2). We implemented additional interventions (PDSA #5 and #6) to augment gains and solidify culture. Wide variability during the baseline period was due to the large number of nurses performing the reassessments and the lack of standardized education on documentation.

**Fig. 3.**
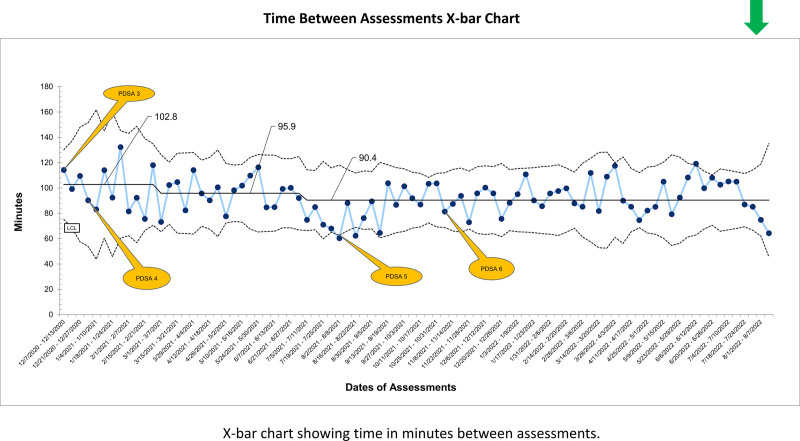
Time between assessments X-bar chart. X-bar chart showing time in minutes between assessments.

The mean time to complete the reassessment was 102.8 minutes, which decreased to 90.4 minutes (Fig. 3). We observed two centerline shifts. From 11/24 to 12/1, we sent 47 text messages and then 47 emails from 12/11/2020 to 4/28/2021. The emails highlighted barriers more effectively, as the nurses answered them more often than text messages (PDSA# 3). Barriers to compliance were high workload and patient acuity. Due to bedside nurse turnover and many nurses from other units, in-person education helped those new to our unit (PDSA# 4).

**Fig. 4.**
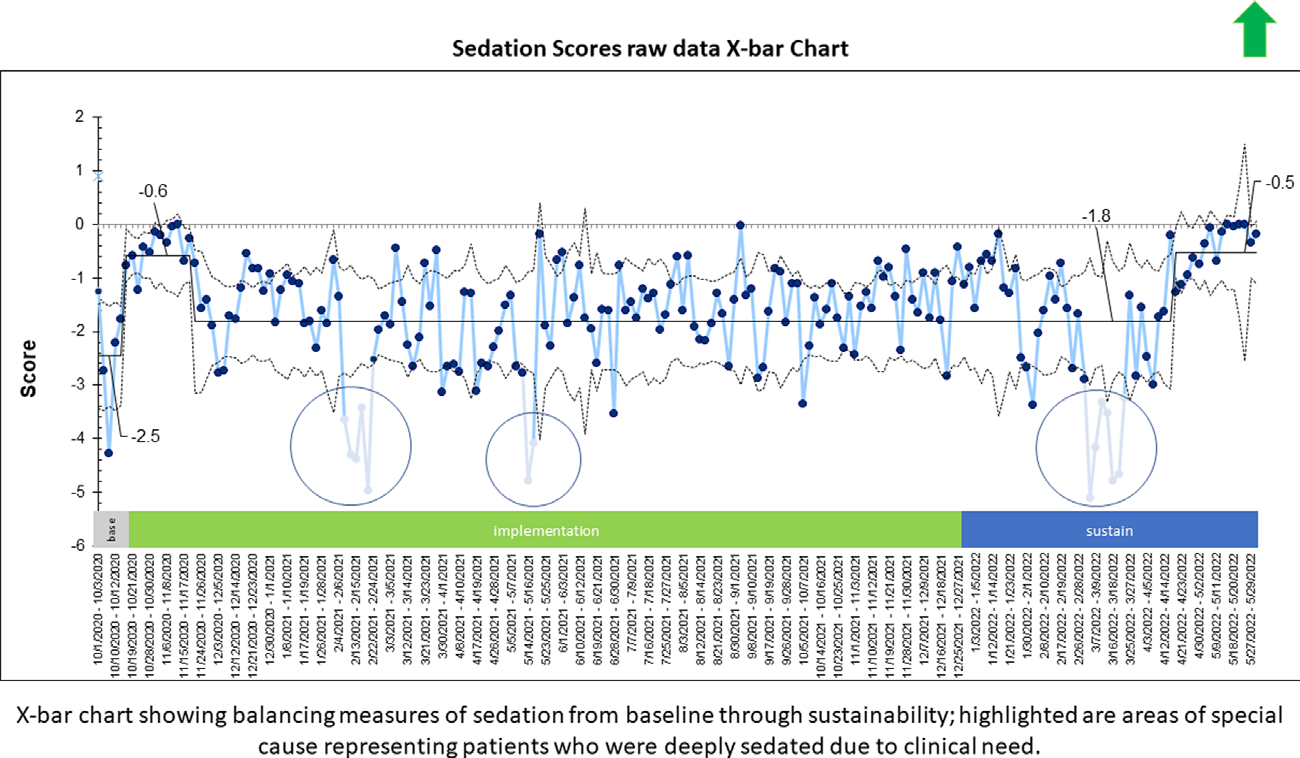
Sedation scores raw data X-bar chart. X-bar chart showing balancing measures of sedation from baseline through sustainability; highlighted are areas of special cause representing patients who were deeply sedated due to clinical need.

The balancing measure of oversedation was measured by the mean sedation score per month (negative scores) on N-PASS before and after implementation and during the sustain phase. Sedation scores had a mean of -2.5 at baseline, -0.6 during the intervention period, and -1.7 by the end. These results remained steady during the sustain phase (Fig. 4). We saw a third shift in the last four months of the sustain period to -0.5 and identified at least three special causes. These special causes occurred with four patients who needed deep sedation while on ECMO, high-frequency oscillator ventilation, and end-of-life care. After removing these four patients with special causes, the control limits narrowed, but they did not change the overall outcome.

## DISCUSSION

Published data show that pain reassessment is not frequently performed across the neonatal, pediatric, and adult populations. Studies show only 15%–77.8% compliance with reassessments in pediatric populations in transitional care, pediatric, neonatal intensive care, and pediatric intensive care unit settings^,11,19–22^ and lower than 41%–94.9% in adult hospitals.^14,23^ As the neonatal population is nonverbal, clinicians rely on objective assessments such as the N-PASS for effective pain management. This information helps clinicians understand if the interventions to mitigate pain are effective. During the implementation of this initiative, we improved neonatal pain reassessment in the NICU through reminders, education, and changes in documentation from 50% to 79%. Margonary et al.^11^ showed no change in pain reassessment after implementing education in a few patients in a transitional care setting, but their interventions did not focus on reassessment specifically. As a result, their study showed an increase in the total number of patients requiring pain reassessments (denominator), but there was no change in those who received it. Similar to our study, Habich et al showed improvement in reassessment from a baseline of 30%–69.4% by implementing comprehensive pain education and developing a guideline.^24^ Reavey et al, in their study of N-PASS implementation in the NICU, noted that reassessment was performed only 30% of the time and 47% after two years despite education and reminders.^22^ Similarly, Aukes et al noted that only 31% of reassessments occurred after a high pain score on the COMFORTneo scale.^15^ Our study is the first study of pain reassessment in the NICU that has shown improvement beyond published rates and sustained for 6 months after implementation.

The strength of our approach was that we confined our intervention to all patients with high scores who were highly in need of pain relief (N-PASS score ≥4). Furthermore, like Nahum et al, we believe a real-time report through the trigger system allowed us to identify noncompliant cases and increase awareness with the nursing staff.^23^ Additionally, we prospectively elicited input into compliance failures by debriefing, enabling us to detect improvement areas proactively.

Compliance with the 60-minute timeframe for reassessment was 79%, with an average of 90.4 minutes overall, down from 102 minutes. A previous study in an adult outpatient clinic showed a compliance rate of 38% with reassessment within 30 minutes of opioid administration.^24^ Our study has shown improvement in compliance and reduced response time. Reasons for noncompliance were mainly forgetting to document. Erdek and Pronovost have previously classified adverse pain management events as either failure to document or manage pain.^25^ In our case, nurses performed the N-PASS and managed pain but forgot to document it. Like Gordon et al,^17^ high workloads and high acuity significantly contributed to the failure to document reassessments timely. These findings also align with Deldar et al’s^26^ qualitative study of barriers to assessing nonverbal patients. It mentions that nurses viewed pain assessment as a “forgotten priority” or that they were saddled with a heavy workload and time limitations.^17,27^ When nurses had no prior experience with assessment scales, they deemed them unimportant. Similarly, the nurses did not perform the assessment when the physicians did not ask.^26,28^ In the case of new nurses, they may also have other learning priorities.^28^ The strong culture of pain control in our NICU, use of standardized assessments, documentation in the EHR, robust nursing education program, and discussion of the pain score on daily rounds contributed to the high baseline rate of reassessment compared to other units. Still, workload issues, time management, and reliance on tachycardia alone to detect pain could have contributed to the 80% upper compliance limit. Similar to previous studies, noncompliant nurses accepted the individual unmasked feedback^28–31^ as we provided it in a nonthreatening confidential manner, and we did not report it to managers.

The improvement team relied on technology and used an existing trigger program to develop on-demand reports of compliance and modification of the EHR when documenting agitation. Although improved, we still need to refine the system. Studies have shown that using the EHR to audit improves documentation compliance in other screening programs,^28,31^ but success hinges on periodic reinforcement. Wissman et al reported using a similar method of sending specific reports to nurses about their performance on pain reassessments in the emergency room. They confirmed that this method was useful in increasing their reassessment rates from 36.2% to 62.3%.^22^ Similarly, Ho et al and Gordon et al instituted weekly dashboards, provided education, addressed documentation barriers, and developed noncompliance user dashboards.^17,31^ Unlike Wissman’s study,^22^ in our project, the presence of an automated report allowed us to monitor all patients in the unit, not just a select few and decreased the burden of chart review. Similarly, Reavey et al.^21^ used a similar solution of alerts and decision support to address “forgetfulness.” Cline^32^ suggested that individual reporting of performance also increases nurse accountability. A systematic review of methods to increase documentation compliance in acute care settings noted that frequent documentation audits, personal feedback, and use of the EHR were the best techniques to achieve reliable and meaningful compliance rates of 70% or more.^30^

This study demonstrated sustained improvement over several months, even with nursing turnover, through continuous learning cycles embedded in our educational curriculum. Lieow et al suggested this approach to sustain compliance with delirium scoring in the adult ICU.^28^ Additionally, direct leadership involvement through continuous bedside coaching, clear accountability, and goal alignment is a means of sustainment.^17^ We presented the project and the automated report to the hospital pain committee to widen its impact. Two other units adopted the trigger report to monitor compliance.

The limitations of this study were that it was a single-center study, and a team member had to retrieve reports manually (author: SI) weekly. The team is now looking to move to a less frequent reporting system that is more sustainable. The project attempted to involve parents in postoperative pain management early on, but we did not pursue this intervention actively due to low parent engagement. Aukes et al suggested theoretically that a goal of 100% reassessment after a readjustment or pharmacological intervention could be set.^15^ In this case, we elected a goal of 75%, as this would most likely be achievable within our current culture. There was variability in the balancing measure (sedation scores), likely due to patient variability. However, it was below the light sedation range (−5 to −2) for most of the study after interventions; therefore, it was not clinically significant.

## CONCLUSIONS

Clinical teams should consistently reassess pain after an initial high score in neonates to manage pain adequately in this unique patient population. Changing culture through persistent reminders should be performed until behaviors change. After that, pursuing automation and continuous education for 6–9 months will maintain high performance. Future studies should assess the effect of applying these interventions in other units with pediatric surgical and nonsurgical patients, the effect of transparent data reporting (visible to all units within the hospital), and the contribution of workload and acuity on compliance rates.

## ACKNOWLEDGMENTS

The authors thank Dr. Billie Lou Short and Dr. Rahul Shah for their support and insight in reviewing this article and their mindful suggestions.

## DISCLOSURE

The authors have no financial interest to declare in relation to the content of this article.

## Supplementary Material


