# Supplementary figures and images for: Improving Documentation of Pain Reassessment after Pain Management Interventions in the NICU
Source: Pediatr Qual Saf. 2023 Sep 28;8(5):e688. doi: 10.1097/pq9.0000000000000688 (PMC10538901; doi:10.1097/pq9.0000000000000688)

Supplemental  
Figure 1

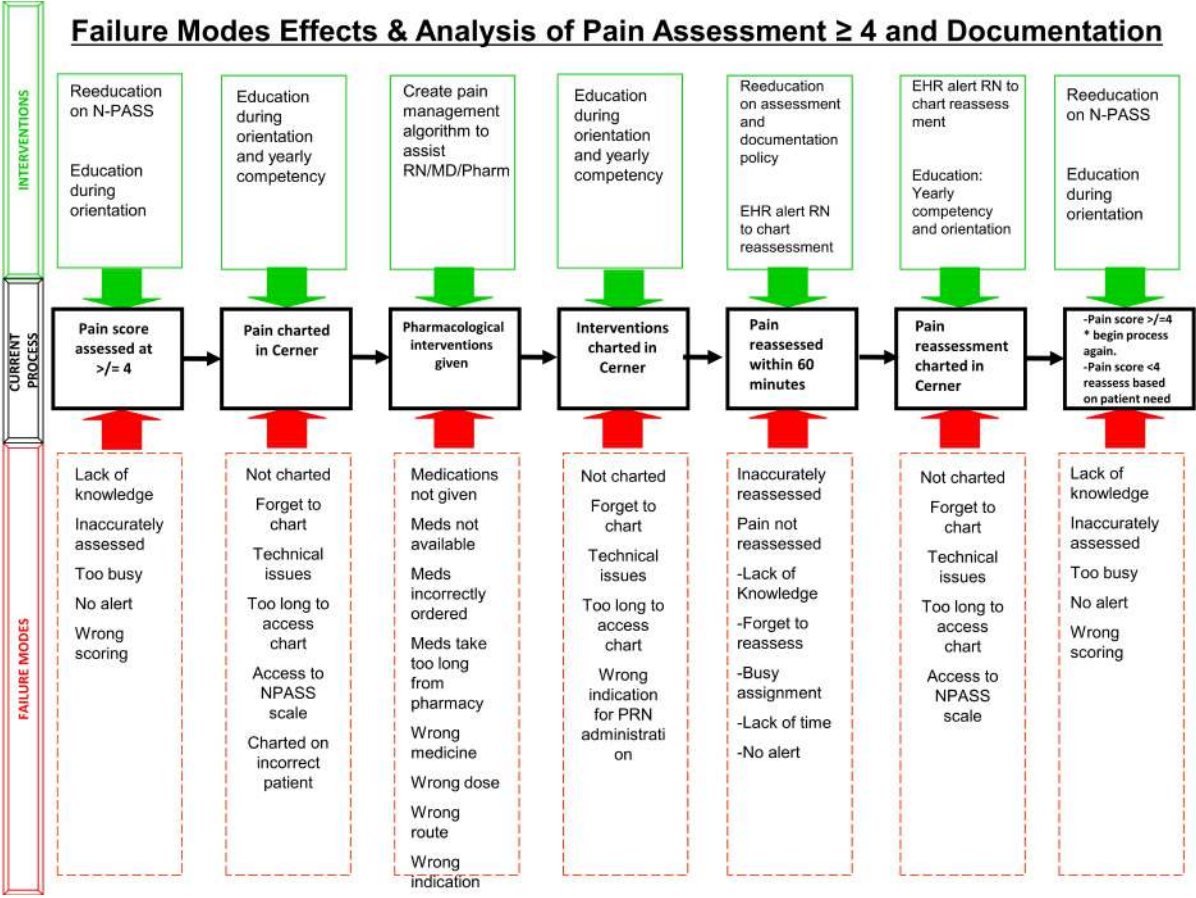

Supplement: Supplementary file 1 [file pqs-8-e688-s001.pdf]
