# Supplementary material for: Improving Documentation of Pain Reassessment after Pain Management Interventions in the NICU
Source: Pediatr Qual Saf. 2023 Sep 28;8(5):e688. doi: 10.1097/pq9.0000000000000688 (PMC10538901; doi:10.1097/pq9.0000000000000688)
Supplement: Supplementary file 2 [file pqs-8-e688-s002.pdf]

Supplemental Figure 2

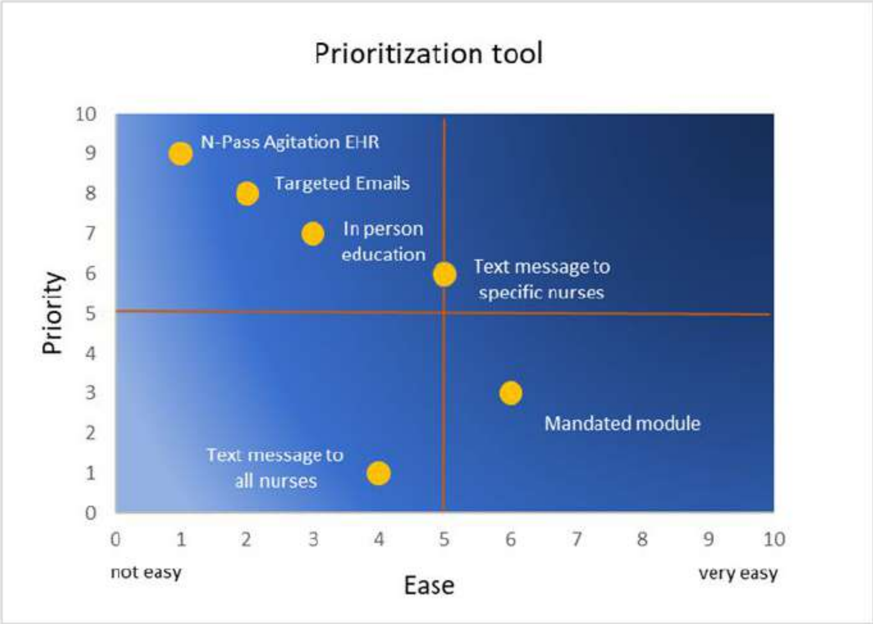

Prioritization tool showing priority scale and ease of implementation of each intervention.
